# A Case of Addison's Disease

**Published:** 1885-12

**Authors:** James E. Prichard

**Affiliations:** Bristol


### A CASE OF ADDISON'S DISEASE. By JAMES E. PRICHARD, B.A., M.B. Oxon., Bristol.

The patient was a man fifty-nine years of age, whose health began to fail twelve years ago; since which time he suffered from occasional attacks of great weakness, accompanied by certain symptoms of faulty digestion; viz., complete loss of appetite, very furred tongue, vomiting, constipation, and sometimes slight jaundice. His wife also told me that she had noticed a brown rash about his forearms, which disappeared as he got better.

I saw him first on the 6th of January, 1885. His present attack began, he told me, just before Christmas. He was confined to bed, and excessively weak, so that moving about, or even talking, seemed almost too great an effort for him. Also he was very emaciated, and had, he thought, been losing flesh rapidly during the last month. His skin was dry and wrinkled, and of a peculiar pallor. Along the knuckles of both hands there was a little irregular brown mottling, but no bronzing elsewhere that I then detected. The conjunctivæ were slightly jaundiced.

Physical examination of his heart and lungs revealed nothing. The pulse was slow and compressible, between 60 and 70, but quite regular.

With regard to the digestive organs, he complained of complete loss of appetite and frequent vomiting. His tongue was furred and his bowels confined. Owing to his being so thin, it was easy to feel the abdominal viscera. The liver seemed to be lower down than usual, but its area of dulness was rather diminished than increased. Firm pressure over this region gave him slight pain, but he complained of no pain or tenderness elsewhere. I could make out no enlargement of the spleen. There was no albumen or sugar in the urine.

I saw him frequently during the course of his illness, but noted very little change in his symptoms. Sometimes he felt a little stronger, and during March came downstairs, and even walked out as far as his garden-gate. The jaundice disappeared, and the vomiting became less troublesome, but never quite ceased, and his appetite improved. However, at the end of March he had a relapse, and became rapidly so weak that he was unable to get out of bed, or even to sit up without assistance. Just before death he became a little incoherent in his speech, and died quietly on the 17th of April, with no further development of his symptoms.

Dr. Shingleton Smith saw this patient with me shortly before his death, and to him is due the credit of pointing out the nature of the case; for until then I thought I had to deal with a case of malignant disease in some way involving and displacing the liver.

On the 18th of April I made a *post-mortem* examination. I then noticed that, besides the bronzing on the knuckles, there were a few small irregular patches on the inner aspect of the thighs and on either side of the penis. These marks were very symmetrical in shape and position. The bladder was sacculated in a peculiar way. The heart was very small and pale, and indeed all the organs were pale, and almost entirely devoid of fat. I did not see any pigmentation upon any of the serous membranes, or on other parts of the body, beyond those I have mentioned. All the other organs, with the exception of the right supra-renal capsule and the vessels forming the cœliac axis (which I exhibited to the Society), were apparently healthy. The right suprarenal capsule is little more than a small empty sac. Its investing membrane is slightly thickened, and was so firmly adherent at the top to the surrounding structures that it was torn in removing it, and the contents which were fluid escaped. The left capsule does not seem to be affected. I have had one or two opportunities since of examining these organs in other bodies, and I think the left capsule is slightly more pigmented and a little smaller than usual. The vessels forming the cœliac axis are curiously dilated into pouches, and their coats separated by calcareous plates.

Under the microscope there appears a little increase in the fibrous tissue, and here and there a few fat cells towards the central portion of the gland. All that can be said is that the capsule is atrophied.

This, then, is a case of uncomplicated Addison's disease; and its symptoms, viz., the great prostration, the vomiting, the general pallor of skin, and the pigmentation, were, I think, typical, and, with the exception of the last, well marked.

There are, however, one or two points to which I wish to refer. Firstly, my patient was of more advanced age than the subjects of this disease generally are. In the cases recorded by Addison himself, three only were above the age of fifty, and in two of the three one capsule alone was affected. The ages of the rest were below forty. In the present instance, also, the right capsule alone is very seriously affected. This fact may, perhaps, also explain the very chronic course which the case ran, and also its recurring exacerbations and remissions.

Then as to the anæmia. This was not a very prominent feature in the case. My patient never complained of any of those symptoms which we usually associate with anæmia. There was no breathlessness upon exertion, no palpitation, nor any abnormal condition of spleen or thyroid; so that I am inclined to think that the colour of the skin is due to a general pigmentary change rather than to any deficiency in blood corpuscles, or any alteration in the relative numbers of white and red. There was, at any rate, no increase in the proportion of white corpuscles in this case. The conjunctivæ are said to have a "pearly" appearance, due, I believe, to contrast with the more deeply pigmented skin, and not to any intrinsic whiteness of the conjunctivæ themselves, just as the whites of negroes' eyes are much more noticeable than those of white men.

There is one point in the pathology of Addison's disease to which I wish to draw your attention, and that is the nature of the lesion in the capsules themselves. It is maintained by some that this is specific, and certain of our text-books even define Addison's disease as "tubercular disease of the supra-renal capsules." In looking through Addison's own cases, I was struck, not at all by the uniformity, but by the great variety of the causes which gave rise to the symptoms. In several it was cancer, in one inflammation, in one tubercle, and in another an effusion of blood, or "kind of apoplexy," as he describes it, of the gland. In this case there is certainly no evidence that the disease is tubercular. It corresponds to the description given by Dr. Wilks at a meeting of the Pathological Society in May last, at which this subject was discussed; viz., "a disease analogous to cirrhosis of the kidney or liver, accompanied by a cell growth of inflammatory type." At that meeting Dr. Bristowe, the President (from an earlier edition of whose work I have quoted the definition of the disease), said: "It appeared that the theory that the disease is tubercular is incorrect, and that the symptoms might be produced by any destructive lesion of the capsules;" and that, I think, must have been the conclusion at which Addison arrived, and to which the balance of evidence seems to incline.

In bringing this subject of Addison's disease before the Society, I purposely avoided any allusion to the Sympathetic; and for this reason, that though no doubt the part which the Sympathetic has to play in the production of the outward and visible signs of the disease is most interesting and important, yet it is still far from clear that there is always a structural change in the semi-lunar ganglia or the nerve-tissues in the neighbourhood, whereas it is scarcely open to doubt that some sort of morbid process occurs in the capsules themselves. I preferred wading in the shallows, where I could make out something of the objects at the bottom, to floundering about out of my depth in the sea of Sympathetic pathology.

